# The effect of Xenin25 on spontaneous circular muscle contractions of rat distal colon in vitro

**DOI:** 10.14814/phy2.14752

**Published:** 2021-02-18

**Authors:** Yuko Kuwahara, Ikuo Kato, Toshio Inui, Yoshinori Marunaka, Atsukazu Kuwahara

**Affiliations:** ^1^ Department of Molecular cell Physiology Graduate School of Medical Science Kyoto Prefectural University of Medicine Kyoto Japan; ^2^ Research Unit for Epithelial Physiology Research Center for Drug Discovery and Pharmaceutical Development Science Research Organization of Science and Technology Ritsumeikan University Kusatsu Japan; ^3^ Department of Medical Biochemistry Kobe Pharmaceutical University Kobe Japan; ^4^ Saisei Mirai Clinics Moriguchi Japan; ^5^ Research Institute for Clinical Physiology Kyoto Industrial Health Association Kyoto Japan

**Keywords:** enteric nervous system, intestinal motility, smooth muscle cell, Xenin25

## Abstract

Xenin25 has a variety of physiological functions in the Gastrointestinal (GI) tract, including ion transport and motility. However, the motility responses in the colon induced by Xenin25 remain poorly understood. Therefore, the effect of Xenin25 on the spontaneous circular muscle contractions of the rat distal colon was investigated using organ bath chambers and immunohistochemistry. Xenin25 induced the inhibition followed by postinhibitory spontaneous contractions with a higher frequency in the rat distal colon. This inhibitory effect of Xenin25 was significantly suppressed by TTX but not by atropine. The inhibitory time (the duration of inhibition) caused by Xenin25 was shortened by the NTSR1 antagonist SR48692, the NK1R antagonist CP96345, the VPAC2 receptor antagonist PG99‐465, the nitric oxide‐sensitive guanylate‐cyclase inhibitor ODQ, and the Ca^2+^‐dependent K^+^ channel blocker apamin. The higher frequency of postinhibitory spontaneous contractions induced by Xenin25 was also attenuated by ODQ and apamin. SP‐, NOS‐, and VIP‐immunoreactive neurons were detected in the myenteric plexus (MP) of the rat distal colon. Small subsets of the SP‐positive neurons were also Calbindin positive. Most of the VIP‐positive neurons were also NOS positive, and small subsets of the NK1R‐positive neurons were also VIP positive. Based on the present results, we propose the following mechanism. Xenin25 activates neuronal NTSR1 on the SP neurons of IPANs, and transmitters from the VIP and apamin‐sensitive NO neurons synergistically inhibit the spontaneous circular muscle contractions via NK1R. Subsequently, the postinhibitory spontaneous contractions are induced by the offset of apamin‐sensitive NO neuron activation via the interstitial cells of Cajal. In addition, Xenin25 also activates the muscular NTSR1 to induce relaxation. Thus, Xenin25 is considered to be an important modulator of post prandial circular muscle contraction of distal colon since the release of Xenin25 from enteroendocrine cells is stimulated by food intake.

## Graphical abstract

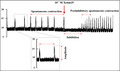

## 1 INTRODUCTION

GI motility is generated by a complex interaction of enteric motor neurons, enteric glia, smooth muscle cells (SMCs), interstitial cells of Cajal (ICCs), PDGRFRα^+^ cells, and muscularis macrophage (Muller et al., 2014; Rao et al., 2017; Sanders et al., 2008, 2016; Wood, 1972). Some of the main functions of the colon are the formation of fecal bulk by water and electrolyte absorption, and the storage of fecal bulk until defecation. Colonic motility disorders are associated with numerous clinical conditions, such as constipation, irritable bowel syndrome, inflammatory bowel disease, and Hirschsprung disease (Barbara et al., 2004; Edery et al., 1994; Törnblom et al., 2002; Wahnschaffe et al., 2001). However, detailed effects of Xenin25 secreted after food intake on the colonic spontaneous circular muscle contractions are still unclear.

The appropriate intestinal motor pattern is controlled by cholinergic motor neurons and nonadrenergic noncholinergic (NANC) inhibitory neurons in the myenteric plexus (MP) (Bennett, 1997). VIP and NO are considered the primary inhibitory NANC neurotransmitters (Keef et al., 1993, 2013; Sanders & Ward, 2019). In addition to NO and VIP, ATP is a cotransmitter in NANC inhibitory neuromuscular transmission (Burnstock, 2012; Burnstock & Novak, 2012). In addition to inhibitory and excitatory motor neurons, the enteric nervous system (ENS) contains intrinsic primary afferent neurons (IPANs) and several interneurons. These enteric neurons form synapses with each other, and on many effectors in the gut wall. The contraction and relaxation of gut smooth muscle are mediated by enteric neurons, and ICC acts as intermediary cells between the neurons and the smooth muscle (Sanders et al., 2016). Dysfunction at any level of this system may potentially disrupt normal motor function.

Xenin belongs to the xenopsin/neurotensin family and is the mammalian counterpart of the amphibian octapeptide xenopsin (Fuerle et al., 1992). This peptide consists of 25 amino acids and was named Xenin1‐25 (Xenin25). Xenin25 is structurally related to the hypothalamic and ileal peptide neurotensin, and the amino acid sequences of the biologically active C‐termini of xenin and neurotensin are structurally related (Fuerle, 1998; Fuerle et al., 2002). The receptors involved in xenin‐ or NT‐induced longitudinal muscle relaxation of rat ileum are coupled to apamin‐sensitive Ca^2+^‐activated K^+^ channels (Clemens et al., 1997). Xenin acts on the neurotensin receptor (Fuerle, 1998). Furthermore, increases in Cl^−^ and HCO_3_^−^ secretion induced by Xenin25 are mediated by the NT receptors in the enteric nervous system (Kaji et al., 2017; Kuwahara et al., 2019). Xenin25 plasma concentrations increase in peripheral circulation after food intake (Fuerle et al., 2003). The localization of Xenin immunoreactivity in secretory granules suggests the identification of enteroendocrine cells as the cellular source of circulating Xenin25, and Xenin25 is cosecreted with the incretin hormone glucose‐dependent insulinotropic peptide (GIP) (Anlauf et al., 2000). However, a recent study suggests that Xenin25 is produced by not only GIP‐containing enteroendocrine cells but also other enteroendocrine cells; a subset of glucagon‐like peptide 2‐, cholecystokinin‐, or 5‐hydroxytryptamine (5‐HT)‐containing enteroendocrine cells also produce Xenin25 (Kaji et al., 2017).

In the GI tract, Xenin25 affects various physiological functions by modulating GI motility, inhibiting gastric acid secretion, and stimulating exocrine pancreatic secretion (Chowdhury et al., 2013; Fuerle, 1998; Fuerle et al., 1997). We recently reported that Xenin25 affects intestinal ion transport through the activation of ENS; Xenin25 induces anion secretion through the activation of IPANs and noncholinergic secretomotor neurons in rat duodenum and ileum, respectively (Kaji et al., 2017; Kuwahara et al., 2019). Based on an *in vitro* experiment, Xenin25 has excitatory and inhibitory effects on smooth muscle activity in the guinea pig GI tract; Xenin induces a biphasic motor response of the small intestine with involvement of the neurotensin receptor, cooperating with muscarinic, purinergic, and tachykinin‐related mechanisms (Fuerle et al., 1996, 2002). On the other hand, Xenin inhibits methacholine‐induced smooth muscle contractions in the guinea pig colon (Fuerle et al., 1996). In addition, the isolated rat ileum is relaxed by Xenin through the activation of the neurotensin receptor (Clemens et al., 1997). Furthermore, the Xenin‐induced contraction of the guinea pig jejunum is completely blocked by TTX, whereas the effect of Xenin on the colon is not affected (Fuerle et al., 1996). These results indicate that the effects of Xenin25 on the smooth muscle activities of the small and large intestines are fundamentally different. In light of this, testing the effect of Xenin25 in colonic muscle activity is important for understanding the regulatory mechanism of smooth muscle activity in the colon. However, there are only a few studies on the effects of Xenin25 on colonic muscle activity *in vivo* or *in vitro*. Therefore, the aim of this study was to examine the effect of Xenin25 on spontaneous circular muscle contractions of rat distal colon *in vitro* to understand the action of Xenin25 on colonic function after food intake.

## 2 MATERIAL AND METHODS

### 2.1 Animals and tissue preparation

#### 2.1.1 Tissue preparation

Male Sprague–Dawley (SD) rats weighing 200 ~ 335 g (Shimizu Laboratory Supplies Co. Ltd., Kyoto, Japan) provided with a pellet diet and water *ad libitum* were used for this study. All studies were performed with approval of the Committee for Animal Research of Kyoto Prefectural University of Medicine (M27‐482). The animals received care, and the experiments were conducted according to the guidelines of the committee. The animals were euthanized by terminal exsanguination under deep isoflurane anesthesia, followed by abdominal section. The segment of the distal colon between the entrances of the left branches of the middle colonic artery and the upper side of the rectum was removed, opened along the mesenteric border, and pinned mucosa side down on a silicon rubber‐lined petri dish filled with ice‐cold Krebs‐Ringer solution containing (in mM) 117 NaCl, 4.7 KCl, 1.2 MgCl_2_, 1.2 NaH_2_PO_4_, 25 NaHCO_3_, 2.5 CaCl_2_, and 11 glucose. To obtain four full‐thickness muscle strips (approximately 2 × 10 mm) containing the mucosa and ENS including myenteric plexus from one animal, the tissue of the distal colon was cut along the circular axis. Experiments were performed using four different full‐thickness muscle strips to evaluate the effect of Xenin25 on spontaneous circular muscle contractions. All the experimental procedures were approved by the Ethical Committee for Animal Research of Kyoto Prefectural University of Medicine.

#### 2.1.2 Measurements and data analysis

**FIGURE 1:**
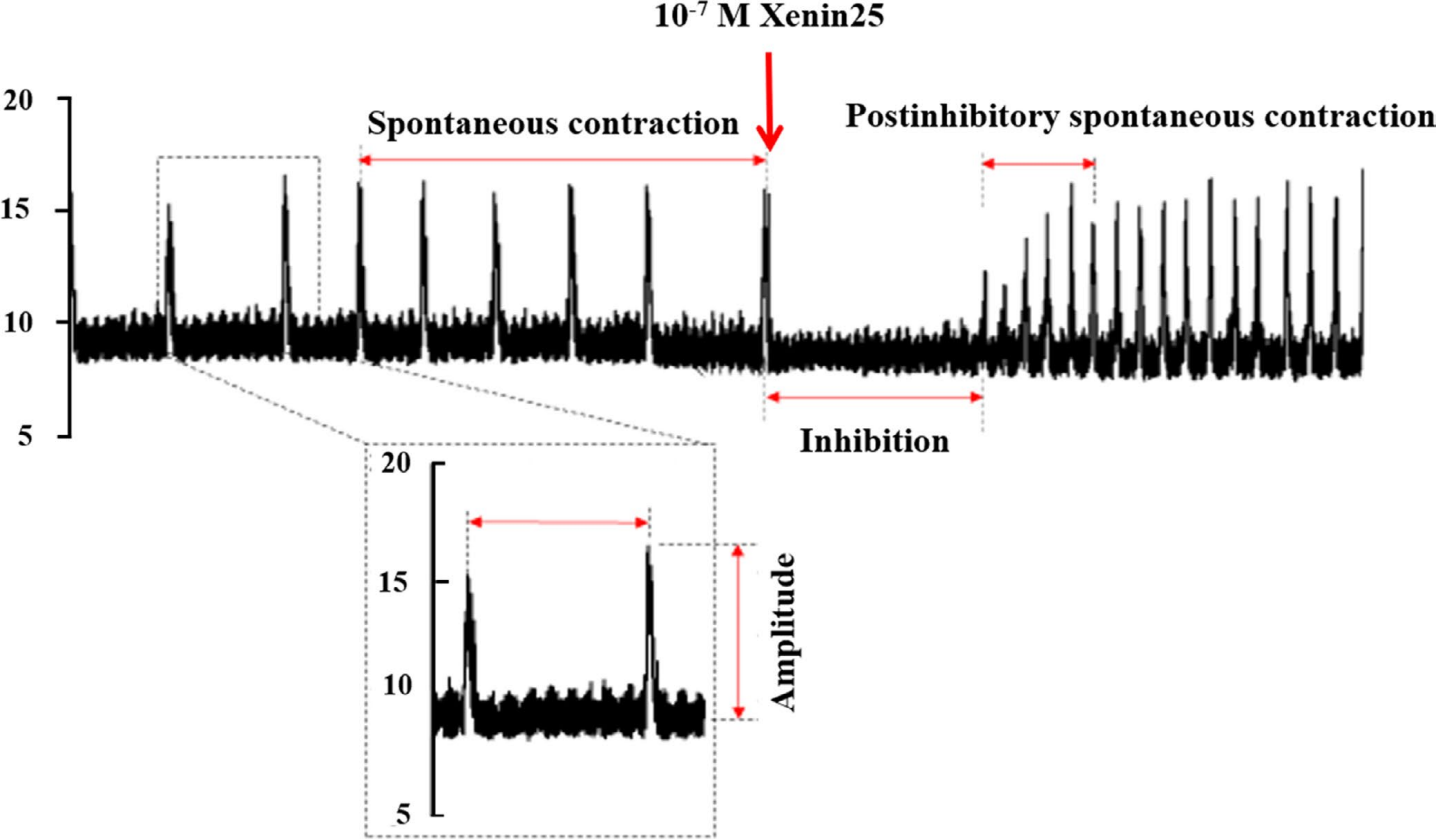
Effects of 10^−7 ^M Xenin25 on the spontaneous circular muscle contractions of the rat distal colon. Xenin25 induced the inhibition followed by postinhibitory spontaneous contractions with a higher frequency in the rat distal colon. The tension of postinhibitory spontaneous contractions gradually recovered but was not stronger than that of preinhibitory spontaneous contractions, and the frequency of postinhibitory spontaneous contractions was higher than that of preinhibitory spontaneous contractions. Xenin25 was applied at the end point of the preinhibitory spontaneous circular muscle contraction (↓). The inhibition time (sec) was quantified as the period between the last peak before the application of Xenin25 and the first peak of the postinhibitory spontaneous contraction. The frequency was quantified as the contractions per minute (cpm), and the tension (mN) was quantified as the amplitude from baseline.

Each circular muscle strip was mounted in a 15‐mL tissue bath containing aerated (5% CO_2_ in 95% O_2_) Krebs‐Ringer solution, maintained at 37 ℃ and connected to an isometric force transducer with silk surgical sutures. A tension of 17 ~ 18 mN was initially applied to the preparations. The circular muscle strips were equilibrated for at least 60 min prior to the application of the test drugs. After equilibration, the effects of pharmacological agents were examined by the addition of these agents to the bathing solution 15 min prior to the addition of Xenin25. The viability of the preparations was confirmed by the presence of spontaneous circular muscle contractions after the equilibration period. Unstable recordings were excluded from further analysis. The changes in the isometric tension were measured via an isometric force transducer (MLT0420 Force Transducer 20 g; AD Instruments, Bella Vista, Australia) and recorded by using the amplifier (AD Instruments) and Mac/Lab4 system (AD Instruments). The custom Mac/Lab program quantified the contractile data at a sampling frequency of 100 Hz. The isometric tension signals were digitized off‐line, and six preinhibitory spontaneous circular muscle contractions, the inhibition time (duration of inhibition), and six postinhibitory spontaneous contractions were analyzed temporally. Xenin25 was applied at the end point of the preinhibitory spontaneous circular muscle contraction (Figure 1). Then, the inhibition time (sec) was quantified as the period between the last peak before the application of Xenin25 and the first peak of the postinhibitory spontaneous contraction (Figure 1). The frequency (cpm) was quantified as the contractions per minute, and the tension (mN) was quantified as the amplitude from baseline (Figure 1). To determine the effects of the test drugs on the Xenin25‐induced inhibition of spontaneous circular muscle contractions, we quantified the inhibition time, changes in the frequency and changes in the tension of postinhibitory spontaneous contractions. The effects of the drugs on the Xenin25‐induced changes in the spontaneous circular muscle contractions are shown as normalized value to the control response for further analysis (Figures 3, 4, 5). The data are expressed as the mean ±SE, and the differences between groups were evaluated by using the Mann–Whitney test for nonparametric variables. The differences were considered significant at *p* < 0.05. The number of experiments used is denoted by “n”, which represents the strip obtained from different rats.

#### 2.1.3 Immunohistochemistry

Four male SD rats weighing 200 ~ 350 g were anaesthetized with isoflurane, and the distal colon was removed. Segments of the distal colon were cut along the mesenteric border, opened, flattened, and pinned mucosa side down. Then, these segments were fixed with 4% paraformaldehyde and 15% saturated picric acid in 0.1 M phosphate buffer (pH 7.4) overnight at 4°C. The fixed tissue segments were washed first with dimethyl sulfoxide (DMSO) and then with PBS. To produce whole‐mount preparations of the longitudinal muscle and myenteric plexus, the submucosal layer, mucosal layer, and circular muscle were peeled off with sharp tweezers under a dissection microscope. The whole‐mount preparations were incubated with 0.3% Triton X‐100 in PBS for 10 min, incubated with Block Ace (Dainippon Seiyaku, Tokyo, Japan) for 30 min at room temperature, and incubated with primary antibodies for 4 days. The primary antibodies used were as follows: rabbit anti‐substance P IgG (1:2000; Yanaihara Inst. Inc., Fujinomiya, Japan), rabbit anti‐NOS IgG (1:250; Yanaihara Inst. Inc., Fujinomiya, Japan), mouse anti‐substance P IgG (1:500; Abcam, Tokyo, Japan), mouse monoclonal anti‐VIP IgG (1:500; Abcam, Tokyo, Japan), rabbit polyclonal anti‐Neurokinin1 receptor (NK1R) IgG (1:50; Abcam, Tokyo, Japan), and mouse monoclonal anti‐Calbindin D‐28 K antibody (1:2000; Abcam, Tokyo, Japan). Antibody specificity was confirmed in a previous study (Arakawa et al., 2002; Goto et al., 2019; Kuwahara et al., 2019; Sorby‐Adams et al., 2019; Tsutsumi et al., 1984). The secondary antibodies used were as follows: Alexa Fluor 594‐conjugated donkey anti‐rabbit antibody (1:2000; Thermo Fisher Sci., Tokyo, Japan), Alexa Fluor 488‐conjugated streptavidin (1:1000; Jackson ImmunoRes. Lab. Inc., West Grove, PA, USA), and biotin‐conjugated goat anti‐mouse antibody (1:150; Sigma‐Aldrich, St. Louis, MO, USA). After being washed with PBS, the whole‐mount preparations were incubated with the biotinylated goat anti‐mouse antibody for 30 min at room temperature. After being washed with PBS again, the whole‐mount samples were incubated with Alexa 488‐conjugated streptavidin and Alexa 594 anti‐rabbit antibodies for 2 hrs at room temperature, washed again with PBS, mounted on glass slides, and coverslipped with fluorescence mounting media. Immunoreactivity was not detected when the primary antibodies were omitted. The immunofluorescence was imaged and captured using AxinoVision (Carl Zeiss GmbH, Jena, Germany).

#### 2.1.4 Peptide synthesis

Xenin25 (MLTKFETKSARVKGLSFHPKRPWIL), the VPAC1 receptor antagonist PG97‐269, and the VPAC2 receptor antagonist PG99‐465 were synthesized using solid‐phase methodology according to the F‐moc strategy with an automated peptide synthesizer (Model Pioneer Thermo Fisher Scientific, Waltham, MA, USA). The crude peptides were purified using reverse‐phase HPLC (Delta 600 HPLC System; Waters, Milford, MA, USA) on a Develosil ODS‐HG‐5 column (2 × 25 cm, Nomura Chemical Co., Ltd, Seto, Japan). The purity of each peptide was confirmed by analytical HPLC and matrix‐assisted laser desorption/ionization time‐of‐flight and mass spectrometry.

#### 2.1.5 Chemicals

A803467, SR48692, CP96345, and MRS2500 were purchased from Tocris Bioscience (Pittsburgh, PA, USA). Apamin was purchased from R&D Systems (Minneapolis, MN, USA). Atropine and tetrodotoxin (TTX) were purchased from Sigma‐Aldrich (St. Louis, MO, USA). ODQ was purchased from Tokyo Chemical Industry (Tokyo, Japan). TTX was dissolved in citrate buffer (pH 4.8), whereas the other drugs were dissolved in DMSO and stored at −20°C. The final DMSO concentration in the bath solution did not exceed 0.1% (15 μl).

#### 2.1.6 Data analysis and statistics

All data are presented as mean ± SE, and *n* represents the number of animals. Statistical analyses were performed using GraphPad Prism 9.0 software (GraphPad Software, La Jolla, CA, USA). Kruskal–Wallis test was used for comparison of more than two groups, followed by Mann–Whitney *U*‐test to compare differences among individual means. A value of *p* < 0.05 was considered statistically significant.

## 3 RESULTS

### 3.1 Effects of Xenin25 on spontaneous circular muscle contractions in rat distal colon

**FIGURE 2:**
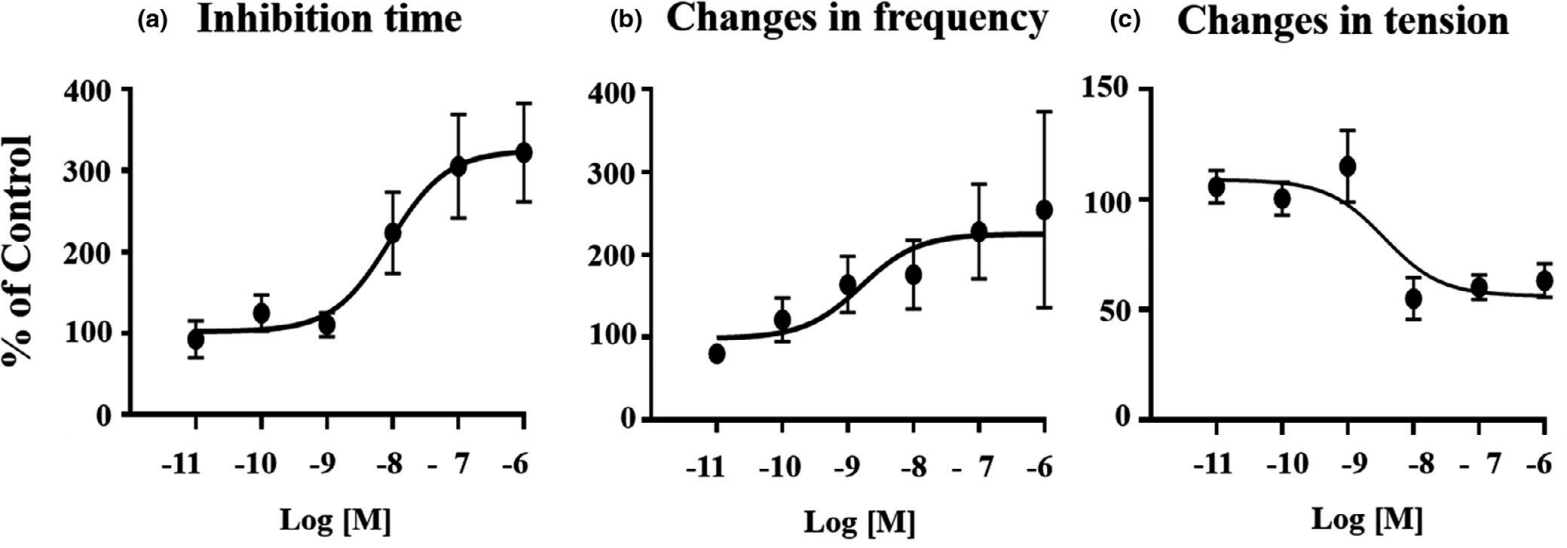
Dose–response curves of Xenin25 on the spontaneous circular muscle contractions in the rat distal colon. The inhibition time (sec) and the change in the frequency of postinhibitory spontaneous contractions were increased in a concentration‐dependent manner, but the change in the tension of postinhibitory spontaneous contractions was decreased adversely. The EC_50_ of the inhibition time was 8.0 × 10^−8 ^M Xenin25, the EC_50_ of the mean change in frequency was 8.8 × 10^−8 ^M Xenin25, and the IC_50_ of the mean change in tension was 8.4 × 10^−8 ^M Xenin25. The values are presented as the mean ± SE (n = 4 ~ 5). The concentration–response curves were analyzed by nonlinear regression curve fitting using GraphPad Prism (v. 9.0; GraphPad Software, San Diego, CA, USA).

**FIGURE 3:**
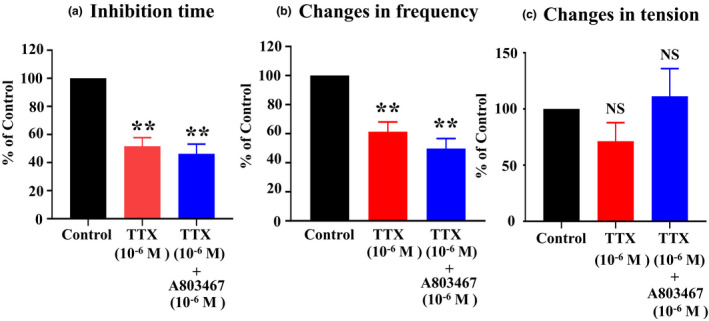
Effects of Na^+^ channel inhibitors on the responses to Xenin25. (a) Inhibition time: The application of 10^−6 ^M TTX (n = 4) or 10^−6 ^M TTX combined with 10^−6 ^M A809367 (n = 8) significantly decreased the inhibition time to the control (10^−8 ^M Xenin25) (*p* < 0.005), but there was no significant difference in the inhibition time observed in response to these two treatments (*p* > 0.05). (b) Changes in frequency: The application of 10^−6 ^M TTX (n = 4) or 10^−6 ^M TTX combined with 10^−6 ^M A809367 (n = 5) significantly decreased the change in the frequency compared to the control treatment (*p* < 0.005), but there was no significant difference in the changes in the frequency observed in response to these two treatments (*p* > 0.05). C. Changes in tension: The application of 10^−6 ^M TTX (n = 4) or 10^−6 ^M TTX combined with 10^−6 ^M A809367 (n = 6) had no effects on the changes in tension induced by 10^−8 ^M Xenin25 (*p* > 0.05).

The spontaneous circular muscle contractions that occurred in the rat distal colon under 17 ~ 18 mN tension were recorded isometrically with a force transducer. The preparations were equilibrated in Krebs‐Ringer solution for at least 60 mins before the drugs were added. The spontaneous circular muscle contractions stabilized at an amplitude of 6.32 ± 0.82 mN (n = 23, mean ±SE) and a frequency of 0.60 ± 0.06 contraction/min. Xenin25 induced the inhibition followed by the postinhibitory spontaneous contractions with a higher frequency in the rat distal colon (Figure 1). The tension of the postinhibitory spontaneous contractions gradually recovered but was not stronger than that of preinhibitory spontaneous contractions (Figure 1). The inhibition time (sec) and the change in the frequency of postinhibitory spontaneous contractions increased in a concentration‐dependent manner, with EC_50_ values of 8.0 × 10^−8 ^M and 8.8 × 10^−8 ^M, respectively (Table 1, Figure 2). In addition, the inhibition time and the changes in frequency reached maximum level at 10^−7 ^M Xenin25. There was a high correlation between the inhibition time (sec) and the change in the frequency of postinhibitory spontaneous contractions induced by 10^−6 ^M (n = 4, R^2^ = 0.99) and 10^−7 ^M (n = 6, R^2^ = 0.90) Xenin25. On the other hand, the change in the tension of postinhibitory spontaneous contractions was decreased in a concentration‐dependent manner, with an IC_50_ value of 8.4 × 10^−8 ^M, as shown in Table 1 and Figure 2. However, there was no correlation between the inhibition time and the change in the tension of postinhibitory spontaneous contractions induced by 10^−6 ^M (n = 4, R^2^ = 7 × 10^−6^) and 10^−7 ^M (n = 5, R^2^ = 0.35) Xenin25. Based on an EC_50_ of the inhibition time and the change in frequency and an IC_50_ of the change in tension, 10^−7 ^M Xenin25 was used for further study, and 10^−8 ^M Xenin25 was used to examine the involvement of ENS on the inhibitory action of Xenin25 on spontaneous circular muscle contractions. (Figure 3).

**TABLE 1:** Effects of Xenin25 on the spontaneous circular muscle contractions of the rat distal colon.

Concenlralion	Inhibition lime (sec)	n	Change in the frequency(%)	n	Change in the tension(%)	n
10^−11 ^M	70.93 ± 10.10	4	80.26 ± 9.51	4	105.82 ± 7.37	5
10^−10 ^M	117.69 ± 27.78	4	121.14 ± 26.39	4	100.43 ± 7.57	5
10^−9 ^M	110.38 ± 14.48	5	164.12 ± 34.23	4	115..11 ± 16.12	5
10^−8 ^M	223.25 ± 50.19	5	175.78 ± 41.84	5	55.08 ± 9.47	5
10^−7 ^M	305.14 ± 63.93	5	249.36 ± 38.01	5	60.24 ± 5.50	5
10^−6 ^M	322.14 ± 60.51	4	254.13 ± 102.51	4	60.89 ± 6.26	4
	EC_50_ = 8.04 × 10^−8 ^M		EC_50_ = 8.80 × 10^−8 ^M		IC_50_ = 8.44 × 10^−8 ^M	

The inhibition time (sec) and the change in the frequency (%) of postinhibitory spontaneous contractions increased in a concentration‐dependent manner, with EC_50_ values of 8.0 × 10^−8 ^M and 8.8 × 10^−8 ^M, respectively (Figure 2a,b). On the other hand, the change in the tension (%) of postinhibitory spontaneous contractions was decreased in a concentration‐dependent manner, with an IC_50_ value of 8.4 × 10^−8 ^M, as shown in Figure 2c. The data are presented as the mean ± SE (n = 4 or 5).

### 3.2 Effects of neuronal blockade and atropine on the response to Xenin25

The neurogenic and myogenic effects of Xenin25 on the motility of the small and large intestines were reported (Fuerle et al., 1996). Therefore, we examined whether the response induced by Xenin25 involved the ENS. In the presence of 10^−6 ^M TTX alone or 10^−6 ^M TTX in combination with 10^−6 ^M A809367 (TTX‐insensitive Na^+^ channel blocker), the inhibition time induced by 10^−8 ^M Xenin25 was significantly decreased to 51.7 ± 6.1% (n = 4, means ± SE) and 47.3 ± 6.0% (n = 8) of the control, respectively (*p* < 0.005, Figure 3a). In addition, the change in the frequency of postinhibitory spontaneous contractions was also significantly decreased to 61.4 ± 6.7% (n = 4) and 49.8 ± 7.0% (n = 5) of the control, respectively (*p* < 0.005, Figure 3b). However, there was no significant difference in the inhibition time or the change in the frequency between 10^−6 ^M TTX alone and 10^−6 ^M TTX in combination with 10^−6 ^M A809367 (*p* > 0.05). The change in the tension of postinhibitory spontaneous contractions was not significantly different from that of the control (*p* > 0.05, Figure 3c). Furthermore, the response induced by 10^−8 ^M Xenin25 was not eliminated by 10^−5 ^M atropine (data not shown). These results suggested that the noncholinergic neuronal mechanism appears to be involved in both the inhibition time and the frequency of postinhibitory spontaneous contractions. However, the neuronal mechanism might not contribute to the change in the tension.

### 3.3 Involvement of noncholinergic neurons in the change in motility induced by Xenin25

**FIGURE 4:**
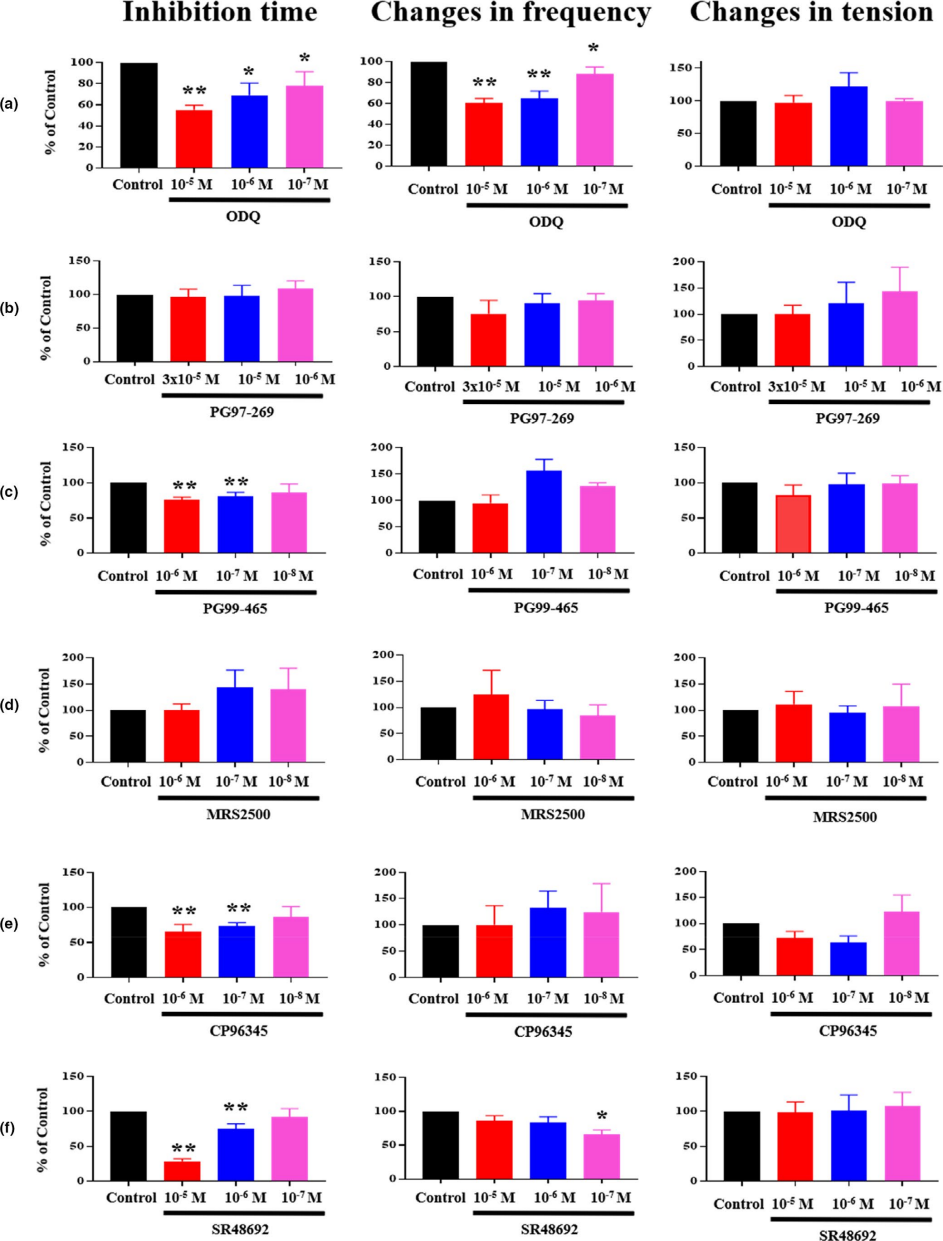
Effects of the selective neural inhibitors on the motility response to 10^−7 ^M Xenin25. Every inhibitor was applied 15 min before the application of 10^−7 ^M Xenin25. A: Guanylate cyclase (NO receptor) inhibitor ODQ (10^−5^~10^−7 ^M), (b) VPAC1 receptor antagonist PG97‐269 (3 × 10^−5^, 10^−5^, 10^−6 ^M), (c) VPAC2 receptor antagonist PG99‐465 (10^−6^~10^−8 ^M), (d) P2Y1 receptor antagonist MRS2500 (10^−6^~10^−8 ^M), (e) NK1R antagonist CP96345 (10^−6^~10^−8 ^M), (f) NSTR1 antagonist SR48962 (10^−5^~10^−7 ^M). The data are presented as the means ±SE (n = 3 ~ 9 and Table 2), and the statistical analysis was performed using the nonparametric Mann–Whitney test. **p < 0.005 and *p < 0.05 compared with the control.

NO, VIP, and ATP are considered noncholinergic inhibitory neurotransmitters (Burnstock, 1972; Burnstock et al., 1970; Fahrenkrug & Emson, 1982; Gillespie & Sheng, 1990; Jin et al., 1993; Keef et al., 1993). Thus, the involvement of the NANC inhibitory pathways in Xenin25‐induced inhibition was examined using various pharmacological inhibitors. The guanylate cyclase inhibitor ODQ reduced the inhibition time and the change in frequency induced by 10^−7 ^M Xenin25 in a concentration‐dependent manner, as shown in Table 2 and Figure 4a. However, ODQ did not affect the change in the tension of postinhibitory spontaneous contractions (Table 2, Figure 4a). These results suggested that NO‐producing neurons appear to be involved in the inhibition time and the change in the frequency of postinhibitory spontaneous contractions but not in the change in the tension.

**TABLE 2:** Effects of the selective neural inhibitors on the motility response to 10^−7 ^M Xenin25.

Pharmacological agents	Inhibition time (%)	n	Change of frequency (%)	n	Change of tension(%)	n
Inhibitor of Guanylate cyclase (sGC)						
10^−5 ^M ODQ	55.26 ± 4.38^**^	5	60.11 ± 4.83^**^	6	97.33 ± 11.04	7
10^−6 ^M ODQ	68.96 ± 11.63^*^	6	64.84 ± 7.10^**^	7	122.19 ± 20.8I	7
10^−7 ^M ODQ	74.50 ± 13.48^*^	3	88.53 ± 6.27^*^	3	99.64 ± 3.93	3
VIP receptor (VPAC1) antagonist						
3 × 10^−5 ^M PG97‐269	96.38 ± 11.67	5	75.34 ± 19.41	5	99.71 ± 17.42	5
10^−5 ^M PG97‐269	98.44 ± 13.81	5	90.76 ± 13.72	3	120.40 ± 36.45	5
10^−6 ^M PG97‐269	109.00 ± 11.50	6	95.05 ± 9.40	6	143.89 ± 45.73	6
VIP receptor (VPAC2) antagonist						
10^−6 ^M PG99‐465	76.24 ± 3.40^**^	9	94.86 ± 15.58	8	82.48 ± 14.31	9
10^−7 ^M PG99‐465	81.01 ± 5.60^**^	5	156.13 ± 21.71	5	98.18 ± 15.55	5
10^−8 ^M PG99‐465	85.71 ± 12.90	5	126.42 ± 7.14	5	99.02 ± 11.24	5
P2Y1 receptor antagonist						
10^−6 ^M MRS2500	99.45 ± 12.49	5	124.16 ± 47.00	5	111.29 ± 24.64	5
10^−7 ^M MRS2500	143.87 ± 32.85	6	96.60 ± 16.93	5	95.35 ± 12.85	5
10^−8 ^M MRS2500	139.80 ± 40.32	6	84.15 ± 20.79	5	107.94 ± 41.83	5
SP receptor (NK1) antagonist						
10^−6 ^M CP96345	64.93 ± 10.71^**^	5	100.01 ± 36.26	4	72.82 ± 12.04	3
10^−6 ^M CP96345	73.06 ± 5.04^**^	5	131.99 ± 32.05	4	63.94 ± 12.40	4
10^−6 ^M CP96345	86.22 ± 15.03	4	124.17 ± 55.10	3	122.49 ± 31.76	3
Neurotensin receptor1 antagonist (NSTR1)						
10^−5 ^M SR48692	28.86 ± 3.32^**^	5	86.87 ± 6.98	4	98.08 ± 15.47	6
10^−6 ^M SR48692	81.64 ± 4.10^**^	4	83.18 ± 9.18	5	100.76 ± 22.76	5
10^−7 ^M SR48692	92.17 ± 11.86	5	65.85 ± 6.71^*^	4	107.23 ± 20.16	5

The data are presented as the mean ±SE (n = 3 ~ 9), and the statistical analysis was performed using the nonparametric Mann–Whitney test. **p < 0.005 and *p < 0.05 compared with the control. The number of experiments used was denoted by “n” that represents number of strips obtained from different rats.

VIP acts via two main G‐protein coupled receptors: VPAC1 and VPAC2 (Dickson & Finlayson, 2009; Harmer et al., 1998; Laburthe et al., 2002). Therefore, VPAC1 and VPAC2 receptor antagonists were used to examine the involvement of VIP in the responses to Xenin25. The VPAC1 receptor antagonist PG97‐269 did not affect the motility response induced by 10^−7 ^M Xenin25 (Table 2, Figure 4b). However, the VPAC2 receptor antagonist PG99‐465 significantly shortened the inhibition time induced by 10^−7 ^M Xenin25 in a concentration‐dependent manner but did not affect the change in postinhibitory spontaneous contractions (Table 2, Figure 4c). Thus, VIP neurons might participate in the inhibition time through VPAC2 receptors, but they are not involved in the postinhibitory spontaneous contractions.

The inhibitory effects of Xenin on the guinea pig GI tract are mediated by a muscular P2 purinoceptor (Fuerle et al., 1996). The purinergic neurotransmission evokes inhibitory neural signals via P2Y1 receptors (Sung et al., 2012). Hence, the P2Y1 receptor antagonist MRS2500 was used to examine the involvement of purinoceptors in the responses induced by Xenin25. MRS2500 did not affect the responses induced by 10^−7 ^M Xenin25 on the spontaneous circular muscle contractions in the rat distal colon, as shown in Table 2 and Figure 4d.

### 3.4 Involvement of tachykinin in the change in motility induced by Xenin25

It has been reported that the response induced by Xenin25 is mediated by SP, which belongs to a group of neurokinins (Feurle et al., 1996). Therefore, the selective NK1R antagonist CP96345 was used to examine the involvement of tachykinins in the responses induced by Xenin25. The inhibition time induced by 10^−7 ^M Xenin25 was significantly attenuated by CP96345; 10^−6 ^M CP96345 and 10^−7 ^M CP96345 shortened the inhibition time (Table 2, Figure 4e). However, CP96345 had little effect on the change in postinhibitory spontaneous contractions (Table 2, Figure 4e). These results suggested that NK1R might be involved in the inhibition time but contribute little to postinhibitory spontaneous contractions.

### 3.5 Involvement of the neurotensin receptor in the change in motility induced by Xenin25

It has been suggested that the effect of Xenin25 on intestinal motility is mediated through NTSR1 due to the sequence similarity between Xenin25 and neurotensin (Clemens et al., 1997; Fuerle et al., 1996, 2002). On the other hand, some biological actions of Xenin25 have been reported to occur without neurotensin receptors (Heuser et al., 2002). Therefore, we also examined whether the motility responses induced by Xenin25 are mediated by NTSR1. The selective NTSR1 antagonist SR48692 was used to confirm the involvement of NTSR1 in the responses induced by Xenin25. SR48692 significantly shortened the inhibition time induced by 10^−7 ^M Xenin25, as shown in Table 2 and Figure 4f. However, all the concentrations of SR48692, with the exception of 10^−7 ^M SR48692, had little effect on the changes in the frequency, as shown in Table 2 and Figure 4f. The change in the tension of postinhibitory spontaneous contractions was not affected by SR48692 (Table 2, Figure 4f). Thus, NTSR1 might be involved in the inhibition time.

### 3.6 Involvement of Ca^2+^‐dependent K^+^ channels in the change in motility induced by Xenin25

**FIGURE 5:**
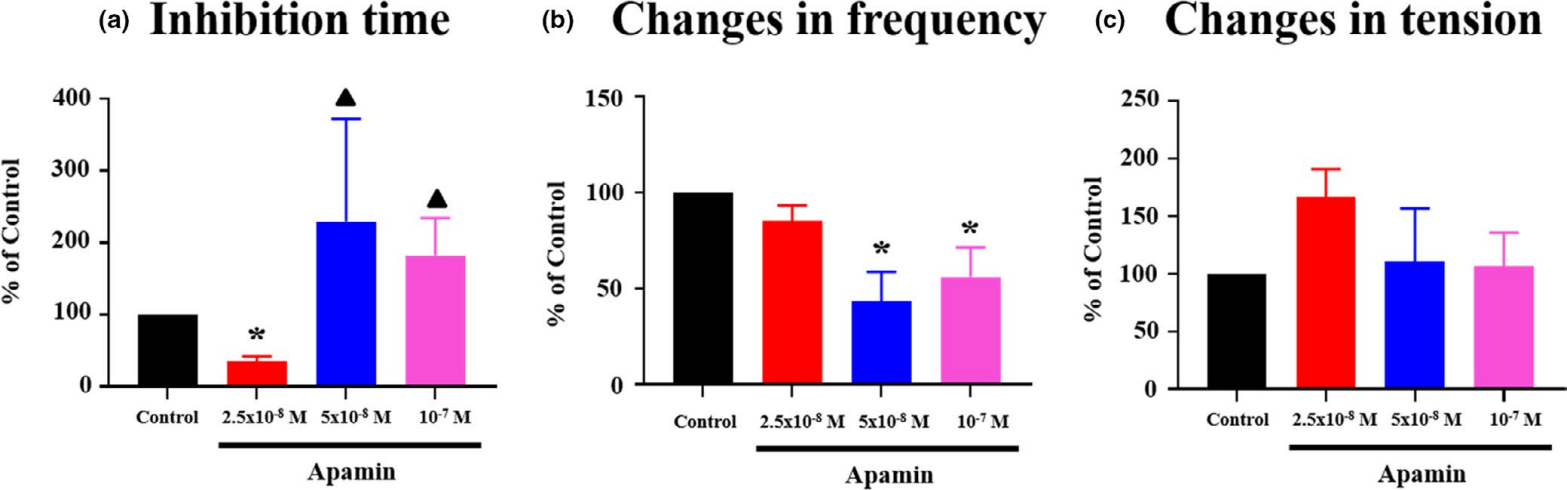
Effects of the small conductance Ca^2+^‐dependent K^+^ (SKCa) channel blocker apamin on the responses to Xenin25. (a) Treatment with 2.5 × 10^−8 ^M apamin significantly shortened the inhibition time (n = 4, *p* < 0.05). (b) Treatment with 5 × 10^−8 ^M and 10^−7 ^M apamin significantly reduced the change in the frequency (n = 3, *p* < 0.05). (c) The change in the tension induced by 10^−7 ^M Xenin25 was not affected by apamin. *p < 0.05 compared with the control. ▲Temporal analysis of the isometric tension signals on the inhibition time was impossible because the tension signals were unstable in the presence of 5 × 10^−8 ^M (n = 3) and 10^−7 ^M (n = 3) apamin. The data are presented as the mean ± SE, and the statistical analysis was performed using the Mann–Whitney test for nonparametric variables.

It has been reported that the inhibitory effects of Xenin on methacholine‐induced guinea pig colonic longitudinal muscle contractions are inhibited by apamin which is a selective small conductance Ca^2+^‐dependent K^+^ channels (sKCa channels) (Clemens et al., 1997; Fuerle et al., 1996). Therefore, we evaluated the involvement of sKCa channels in the response to Xenin25 using apamin. Temporal analysis of the isometric tension signals on the inhibition time was possible only in the presence of 2.5 × 10^−8 ^M apamin because the tension signals were unstable in the presence of higher concentrations of apamin. Apamin (2.5 × 10^−8 ^M) significantly shortened the inhibition time, as shown in Figure 5a. Apamin at concentrations of 5 × 10^−8 ^M and 10^−7 ^M significantly inhibited the change in the frequency of postinhibitory spontaneous contractions (Figure 5b). On the other hand, apamin did not affect the change in the tension of postinhibitory spontaneous contractions (Figure 5c). These results showed that sKCa channels might be involved in the inhibition time and the change in the frequency of postinhibitory spontaneous contractions induced by Xenin25.

### 3.7 Immunohistochemistry of distal colon myenteric plexus

The present results suggested that NTSR1 and NK1 might be involved in the inhibition of spontaneous circular muscle contractions induced by Xenin25 (Figure 4e,f).

**FIGURE 6:**
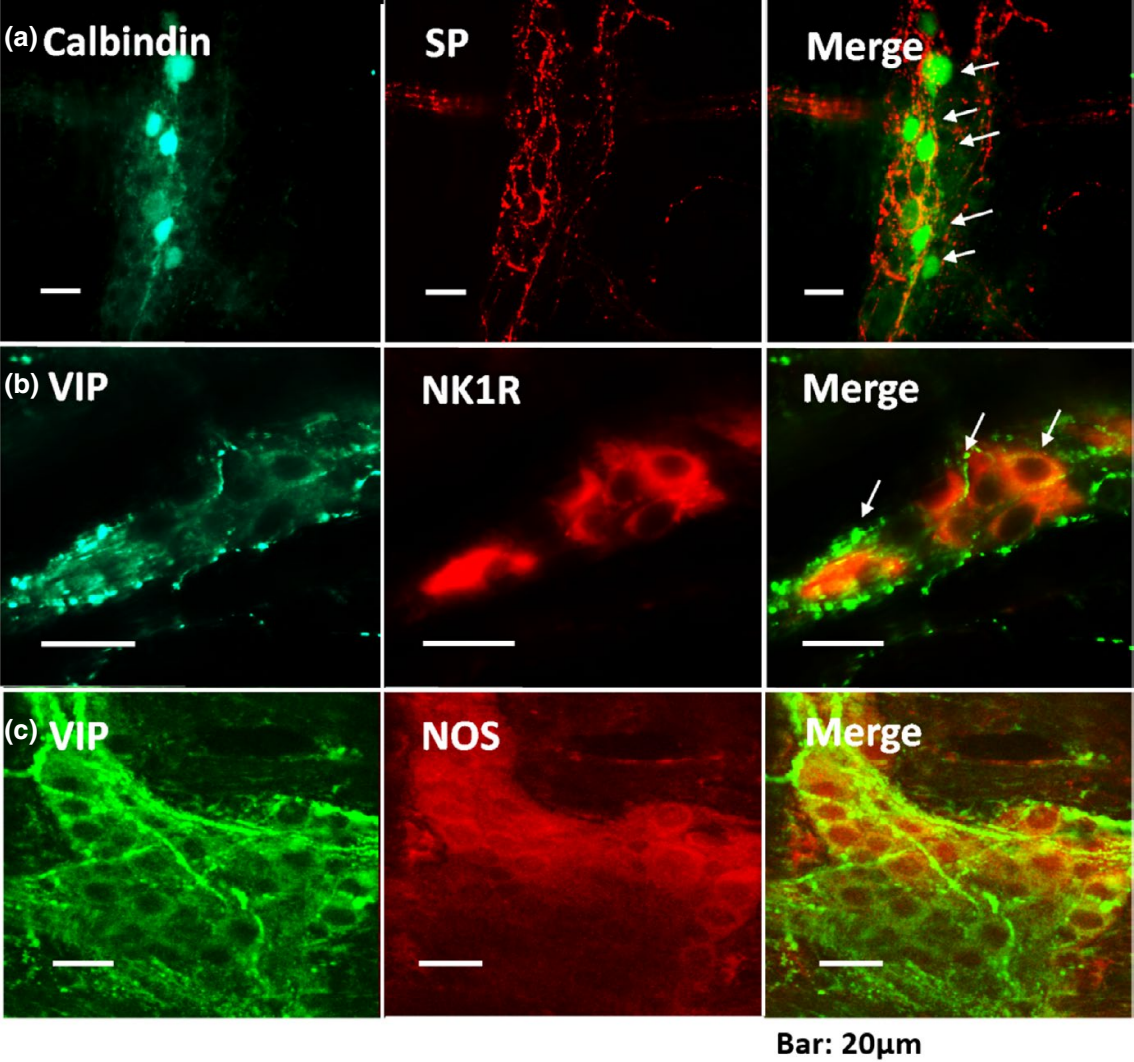
Distribution of the substance P (SP)‐, neurokinin receptor 1 (NK1R)‐, Calbindin‐, vasoactive intestinal polypeptide (VIP)‐, and NO synthase II (NOS)‐positive neurons in the myenteric plexus of the rat distal colon. (a) Double immunostaining for calbindin (green) and SP (red) in the myenteric plexus of the rat distal colon. Note that the immunoreactivity for Calbindin and SP colocalized in a subset of myenteric neurons (arrows). (b) Double immunostaining for VIP (green) and NK1R (red) in the myenteric plexus of the rat distal colon. Note that the immunoreactivity for VIP and NK1R colocalized in a subset of myenteric neurons (arrows). (c) Double immunostaining for VIP (green) and NOS (red) in the myenteric plexus of the rat distal colon. Note that the immunoreactivity for VIP and NOS colocalized in a subset of myenteric neurons (arrows).

We simultaneously incubated the whole‐mount preparations with a polyclonal antibody against SP and a polyclonal antibody against calbindin to examine the relationship between SP‐ and calbindin‐positive nerves. As shown in Figure 6a Merge, SP‐positive neurons exhibited calbindin immunoreactivity. Subsequently, double immunostaining for VIP and NK1R was performed. A large subset of the VIP‐immunoreactive myenteric neurons were also NK1R immunoreactive in the rat distal colon (Figure 6b Merge). Next, double‐immunohistochemistry staining for VIP and NO synthase (NOS) was performed. Many of the VIP‐immunoreactive myenteric neurons in the rat distal colon were also NOS immunoreactive, as shown in Figure 6c Merge. No detectable staining was observed when the antiserum was preabsorbed with the peptide (Table 3).

**TABLE 3:** List of primary antibodies.

Antigen	Source/Reference	Delution	Species
Substance P	Yanaihara, Shizuoka, Japan	1:2000	rabbit antibody
Substance P	Abcam, Tokyo, Japan	1:500	mouse monoclonal antibody
VIP	Abcam, Tokyo, Japan	1:500	mouse monoclonal antibody
NO synthase II	Yanaihara, Shizuoka, Japan	1:250	rabbit antibody
Neurokinin1 receptor (NK1 R)	Abcam, Tokyo, Japan	1:50	rabbit polyclonal antibody
Calbindin D−28 K	Abcam, Tokyo, Japan	1:2000	mouse monoclonal antibody

## 4 DISCUSSION

Xenin25 induced inhibition followed by postinhibitory spontaneous contractions with higher frequency, and the tension of postinhibitory spontaneous contractions was recovered but was not stronger than that of preinhibitory spontaneous contractions (Figure 1). By temporal analysis of the isometric tension signals, the inhibition time and the change in the frequency of postinhibitory spontaneous contractions were shown to increase in a concentration‐dependent manner (Figure 2a,b), but the change in the tension of postinhibitory spontaneous contractions was shown to decrease in a concentration‐dependent manner (Figure 2c). Additionally, there was a high correlation between the inhibition time and the change in the frequency of postinhibitory spontaneous contractions, but there was no correlation between the inhibition time and the change in the tension of postinhibitory spontaneous contractions. It has been shown that inhibition via NANC neurons is followed by postinhibitory rebound contractions at the offset of stimulation (Wood, 1972). Furthermore, neurogenic and myogenic effects of Xenin25 on intestinal motility have been reported (Fuerle et al., 1996). The inhibition time and the change in the frequency of postinhibitory spontaneous contractions were significantly suppressed by TTX (Figure 3a,b), but the response induced by Xenin25 was not eliminated by 10^−5 ^M atropine. In contrast, the change in the tension of postinhibitory spontaneous contractions was not significantly suppressed in the presence of TTX (Figure 3c). Based on these observations, noncholinergic or NANC neurons innervating the circular muscle of rat distal colon appear to be involved in both of the inhibition time and the frequency change, but myogenic activity might be involved in the tension change induced by Xenin25.

The enteric neural circuit involved in the Xenin25‐induced motility response has not been characterized in detail. Hence, we examined the effects of NO, VIP, and ATP, as they are dominant noncholinergic inhibitory neurotransmitters, to test our hypothesis that noncholinergic neurons might be involved in the inhibition time and the change in the frequency of postinhibitory spontaneous contractions. The guanylate cyclase inhibitor ODQ and the VPAC2 inhibitor PG99‐465 led to concentration‐dependent suppression of the inhibition time, and ODQ also suppressed the change in the frequency of the postinhibitory spontaneous contractions in a concentration‐dependent manner (Table 2, Figure 4a,c). The constitutive form of nitric oxide synthase is expressed in ICCs and SMCs (Battish et al., 2000; Gronberg et al., 2011; Lino et al., 2008; Xue et al., 1994), and NO neurons, such as NANC neurons, act on both ICCs and SMCs (Gronberg et al., 2013; Lies et al., 2014; Publicover et al., 1993; Shahi et al., 2014). Furthermore, nitrergic inhibitory neurotransmission in GI muscles is mediated by ICC (Lies et al., 2014; Sanders et al., 2016). In light of these findings and our results, Xenin25 might induce the release of NO to directly and indirectly inhibit spontaneous contractions through ICCs. The band for the *Vipr2* PCR product was detected in the muscle layer of the rat ileum and gastric fundus (Harmer et al., 1998; Kuwahara et al., 2019), and VIP directly relaxed the circular muscle via the activation of VPAC2 receptors on the SMCs in guinea pig ileum and colon (Harmer et al., 2004; Mahavadi et al., 2013). Considering these findings and our results, Xenin25 might induce VIP release to directly inhibit spontaneous contractions via the VPAC2 receptor on SMCs. VIP/nNOS (neuronal isoform of NO synthase) colocalized within inhibitory motor neurons in the GI tract of many species (Humenick et al., 2019), similar to our immunohistochemistry results (Figure 6c Merge), and neurally induced relaxation represents the combined effects of VIP and NO on SMCs (Grider, 1993; Ivancheva et al., 2001). The P2Y1 receptor antagonist MRS2500 and the VPAC1 inhibitor PG97‐269 had no effect on the motility response induced by 10^−7 ^M Xenin25 in the present study (Table 2 and Figure 4b,d). It was reported that the inhibitory effects of Xenin are mediated by the P2 purinoceptor on SMCs (Fuerle et al., 1996), but we could not clearly show the involvement of the purinoceptor. The bands for the *Vipr1* PCR product were detected in the mucosal and submucosal layers of the rat ileum but not in the muscle layer (Kuwahara et al., 2019), so VIP neurons might not be involved the Xenin25‐induced motility response via VPAC1 receptors.

It has been reported that the inhibitory effect of Xenin25 is mediated by apamin‐sensitive sKCa channels (Clemens et al., 1997; Fuerle et al., 1996). Treatment with 2.5 × 10^−8 ^M apamin significantly shortened the inhibition time (Figure 5A), and treatment with 5 × 10^−8 ^M and 10^−7 ^M apamin significantly attenuated the change in the frequency of postinhibitory spontaneous contractions (Figure 5b). Apamin is a noncompetitive, nonspecific antagonist of the nonadrenergic inhibitory transmitter (Maas & Den Hertog, 1979), and it has been used as a blocking agent of sKCa channels (Matsuda & Miller, 2010). Based on these studies and our results, Xenin25 might induce inhibition followed by higher‐frequency postinhibitory spontaneous contractions through the activation of apamin‐sensitive NO‐releasing motor neurons.

The gut is innervated by not only enteric neurons but also several classes of extrinsic neurons (the autonomic nervous system). Extrinsic afferent neurons convey their action potential through TTX‐insensitive Nav1.8 sodium channels, which are rarely observed in enteric neurons (Gautron et al., 2011; Miranda‐Morales et al., 2010). Regarding the inhibition time and the change in the frequency of postinhibitory spontaneous contractions, there was no significant difference between the application of TTX alone and the combined application of TTX and A809367 (Figure 3a,b). Thus, the extrinsic afferent neurons were not involved in the response of the rat distal colonic circular muscle to Xenin25. Some of the effects of Xenin25 on muscle contraction in the rat distal colon have been reported to be mediated by SP, which is released from IPAN endings (Fuerle et al., 1996). Additionally, SP neurons cause an inhibitory response in presynaptic sites and the subsequent stimulation of VIP and NO neurons (Jin et al., 1993). In the present study, the NK1 receptor antagonist CP96345 significantly shortened the inhibition time, but it had little effect on the change in the postinhibitory spontaneous contractions (Table 2, Figure 4E), and SP‐positive neurons also expressed calbindin, which is a marker of IPAN (Figure 6a). Since neurokinin is a mediator of presynaptic sites on NO and VIP‐releasing neurons, the inhibition might be mediated by SP neurons, such as IPAN, but these neurons were not involved in the higher‐frequency postinhibitory spontaneous contractions induced by the offset of the NO neuronal stimuli.

The effects of Xenin on peristaltic activity are blocked by the NTSR1 antagonist SR48692 and are mediated by muscular NTSR1 or neuronal NTSR1 in the MP due to the sequence similarity between Xenin and neurotensin (Clemens et al., 1997; Fuerle et al., 1996, 2002). Localization of NT binding sites on smooth muscle as well as myenteric and submucous ganglia in guinea pig ileum and porcine jejunum was demonstrated (Azriel & Burcher, 2001; Goedert et al., 1984; Seybold et al., 1990). SR48692 at 10^−5 ^M and 10^−6 ^M significantly shortened the inhibition time. However, all the concentrations of SR48692, with the exception of 10^−7 ^M SR48692, had little effect on the change in the frequency of postinhibitory spontaneous contractions induced by the offset of the neuronal stimuli. The inhibition induced by Xenin25 seems to be mediated by both muscular and neuronal NTSR1, but we could not clearly show the involvement of NTSR1 on the change in frequency of postinhibitory spontaneous contractions.

In conclusion, Xenin25 activates the neuronal NTSR1 on SP neurons of IPANs, and the transmitters from the VIP‐ and apamin‐sensitive NO neurons synergistically inhibit spontaneous circular muscle contractions via NK1R. Subsequently, higher‐frequency postinhibitory spontaneous contractions are induced by the offset of apamin‐sensitive NO neurons activation via the interstitial cells of Cajial (ICCs). In addition, Xenin25 also activates the muscular NTSR1 to induce relaxation. These complex effects of Xenin25 on spontaneous circular muscle contraction may contribute the regulation of colonic motility by food intake.

## CONFLICT OF INTEREST

The authors declare no conflicts of interest.

## AUTHOR CONTRIBUTIONS

YK and AK conceived the study, designed the experiments, performed the experiments, and analyzed data. YK and AK drafted the manuscript. YK and AK revised the manuscript. IK made the peptides for use experiments. All authors approved the final manuscript.
